# Elevated serum levels of interleukin-11 and matrix metalloproteinase-9 in myalgic encephalomyelitis/chronic fatigue syndrome

**DOI:** 10.3389/fimmu.2026.1827700

**Published:** 2026-06-05

**Authors:** Baskaran Chinnappan, Duraisamy Kempuraj, Kristina K. Aenlle, Ashley Middleton, Katie S. Day, Sai Puneeth Kothuru, Rhitik Samir Joshi, Nancy G. Klimas, Theoharis C. Theoharides

**Affiliations:** 1 Institute for Neuro-Immune Medicine, Dr. Kiran C. Patel College of Osteopathic Medicine, Nova Southeastern University, Ft. Lauderdale, FL, United States; 2 Miami Veterans Affairs (VA) Geriatric Research Education and Clinical Center (GRECC), Miami Veterans Affairs Healthcare System, Miami, FL, United States; 3 College of Psychology, Nova Southeastern University, Ft. Lauderdale, FL, United States; 4 Department of Immunology, Tufts University School of Medicine, Boston, MA, United States

**Keywords:** IL-11, mast cells, matrix metalloproteinase-9, myalgic encephalomyelitis/chronic fatigue syndrome, serum biomarkers

## Abstract

Myalgic encephalomyelitis/chronic fatigue syndrome (ME/CFS) is a disease of unknown etiology associated with chronic severe fatigue and neurological symptoms, including dizziness, sleep disturbances, cognitive impairment and pain. There are no reliable blood biomarkers available for ME/CFS, possibly due to the lack of specific pathogenesis, even though Epstein-Barr Virus (EBV) has been suspected. We quantified the levels of interleukin-11 (IL-11) in the serum of female ME/CFS patients (n = 40; mean age 51 years) and age- and gender-matched healthy control subjects (n = 38; mean age 43), as well as matrix metalloproteinase-9 (MMP-9) in ME/CFS patients (n = 18; mean age 57 years old) and healthy control subjects (n = 18; mean age 53 years old), using an enzyme-linked immunosorbent assay (ELISA). We hypothesized that mast cells (MC) stimulated by EBV may be involved. MC are unique tissue immune cells that have been implicated in ME/CFS. MC were grown from human umbilical cord blood CD34^+^ stem cells *in vitro* and incubated with recombinant (rEBV) protein, following which the release of MMP-9 was assayed in the cell culture supernatant media by ELISA. There was a significant increase in serum levels of IL-11 and MMP-9 in ME/CFS patients compared to control subjects. MCs stimulated by rEBV protein released a high amount of MMP-9 compared to control cells. In conclusion, IL-11, MMP-9 and MCs may be involved in ME/CFS individuals.

## Introduction

Myalgic encephalomyelitis/chronic fatigue syndrome (ME/CFS) is a disabling complex chronic multisymptom disorder associated with severe fatigue and neurological symptoms, including sleep disturbance, cognitive impairment and dysautonomias (1–5). It is estimated that about 3.3 million people in the United States of America (U.S.A) suffer from ME/CFS (Centers for Disease Control and Prevention (CDC) (6). Its prevalence is about 0.42% among adults, and the females are affected three times more than males (7). The ME/CFS is most common in people between the ages of 40–60 years (6). Multifactoral factors including viral infections and environmental triggers, could contribute to immune dysfunction, neuroinflammation and brain changes involved in ME/CFS pathogenesis (5, 8). In particular, Epstein-Barr Virus (EBV) has been implicated (9–11). There are no effective treatments for ME/CFS because the causes and disease mechanisms are not clearly understood yet (8).

Despite efforts to identify blood biomarkers, except for some proinflammatory cytokines being elevated early in the course of the disease (12), no blood biomarker has been found to be significantly associated with ME/CFS (13). A recent systematic review concluded that all potential ME/CFS biomarkers differed in efficiency, quality, and translatability, with poor reproducibility of findings between studies (1). It was recently reported that there were stress-induced immune signature changes in ME/CFS patients (14).

Interleukin-11 (IL-11) is a proinflammatory, IL-6 family member expressed by many cell types, including astrocytes, monocytes, macrophages, endothelial cells, dendritic cells, neutrophils and damaged cells (15–19). IL-11 has been implicated in several inflammatory and autoimmune diseases (19), including diseases of the nervous system (15, 20), especially with Multiple Sclerosis (MS) (18, 23). IL-11 is also implicated in senescence and aging pathologies, and inhibition of IL-11 can extend mammalian health span and lifespan (20).

Matrix metalloproteinases (MMPs) are enzymes that degrade the components of the extracellular matrix (ECM) development, and implicated in angiogenesis, and the wound healing process in the body (21), but abnormal expression of MMPs leads to loss of normal degradation of ECM and has been implicated in neurodegenerative diseases (21). In particular, MMP-9, a macromolecular zinc-dependent endopeptidase, disrupts neuronal connectivity, disrupting ECM homeostasis and reducing cell-cell adhesion have been reported in ME/CFS (36), thus contributing to neuropsychiatric disorders (22–24). Disrupted ECM homeostasis and reduced cell-cell adhesion have been reported in ME/CFS (25). MMP-9 could be released from microglia (26), but also from mast cells (MC) (27). Interestingly, MC activation disorders are a comorbidity in patients with ME/CFS (28). We recently reported elevated levels of MMP-9 in the serum of Long COVID patients compared to healthy control subjects (29). In fact, ME/CFS and Long COVID share several similar symptoms and pathological characteristics, including immune and inflammatory abnormalities and gene expression signatures (30, 31).

In the present study, we report elevated levels of both IL-11 and MMP-9 in serum from patients with ME/CFS compared to healthy controls, as well as release of MMP-9 from cultured human MCs stimulated by recombinant (rEBV) protein.

## Materials and methods

ME/CFS patients for this study were selected as described previously (32). These patients met the 1996 CDC/Fukuda and 2003 Canadian Case definitions for ME/CFS and were recruited (2007–2012) at the Department of Medicine, University of Miami Miller School of Medicine. Inclusion criteria were post-exertional malaise, prolonged fatigue and tiredness, pain, immunological abnormalities, sleep disturbances and cognitive disorder. Exclusion criteria were the presence of any other active medical conditions, such as diabetes, neuropsychiatric disorders, substance abuse or use of immunotherapeutic agents. This study was conducted in accordance with the University of Miami/Nova Southeastern University (NSU) Institutional Review Board (IRB) approved guidelines (# 20060815).

Serum samples from age-matched healthy female control subjects were obtained frozen from BioIvt Elevating Science (Hicksville, NY, USA) and stored at -80 °C until used for assays. These samples were collected under informed consent and other appropriate regulatory and ethical approvals. The blood samples were collected in serum separation tubes, allowed for 1 hour at room temperature, centrifuged for 10 min at 2000xg in a refrigerated centrifuge and serum was collected, aliquoted and stored at -80 °C until analysis using enzyme-linked immunosorbent assay (ELISA) was performed.

We measured the levels of IL-11 (Catalog #DMP900, BioTechne R&D System, Minneapolis, MN, USA) in the serum of female ME/CFS patients (n=40; mean age 51 years old) and age and gender-matched healthy control subjects (n=38; mean age 43 years old) by ELISA using commercial Kits and microplate reader (FilterMax F5 Multi-Mode Microplate Reader, Molecular Devices), as we have reported previously (29). We also measured serum MMP-9 (Catalog #DY911, BioTechne R&D System, Minneapolis, MN, USA) levels in female ME/CFS patients (n=18; mean age 57 years old) and age and gender-matched healthy control subjects (n=18; mean age 53 years old). The number of samples in ME/CFS patients used to measure IL-11 and MMP-9 are different due to the limited quantities of serum samples available for this study.

### MC culture, stimulation and MMP-9 ELISA

MCs were grown from human umbilical cord blood-derived CD34^+^ cells by incubating with stem cell factor (SCF, 100 ng/ml) and IL-6 (50 ng/ml) in Iscove’s Modified Dulbecco’s Medium (IMDM) for 14–16 weeks, as we have previously reported (33). These MCs were incubated with recombinant EBV protein (rEBV, Abcam, Waltham, MA, USA) at 100 ng/ml or lipopolysaccharide (LPS; ThermoFisher Scientific/Invitrogen, Miami, FL, USA) at 10 ng/ml in 24-well tissue culture plates (1x10^5^ cells/ml media/well) in serum-free media. Then the supernatant media was collected after centrifugation and stored at -80°C freezer until ELISA for MMP-9 (Catalog # DY911, BioTechne R&D System, Minneapolis, MN, USA) using a commercial ELISA kit.

### Statistical analysis

The results are presented as mean ± SEM. Statistical analysis of the data was performed using the Unpaired non-parametric Mann-Whitney test, and one-way analysis of variance (ANOVA) followed by Tukey’s post hoc analysis to determine statistically significant differences between the healthy controls and ME/CFS patients, Tukey’s test protects against type 1 error inflation. All analyses and graphics were performed using GraphPad Prism (Version 10.6.0, GraphPad Software, Boston, MA, USA). Scatter graphs represent individual data for both ME/CFS and healthy control subjects with mean bars. A p-value of less than 0.05 was considered statistically significant.

## Results

**Figure 1:**
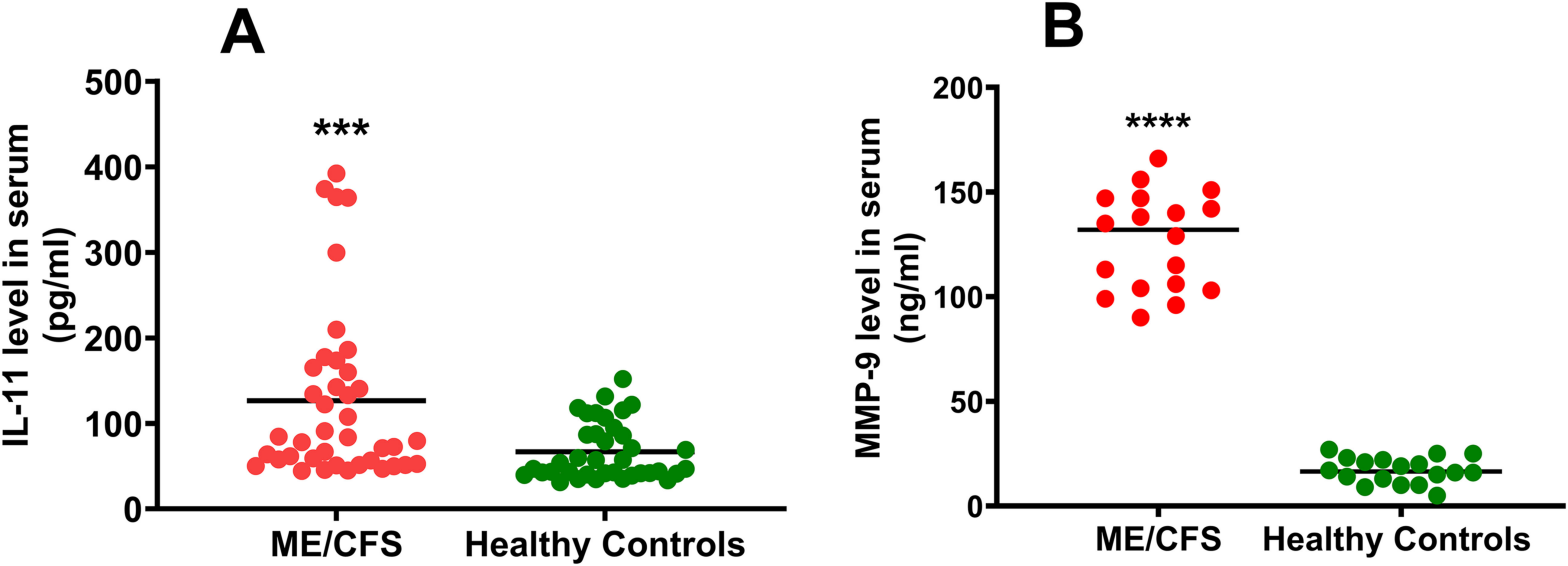
Elevated levels of serum IL-11 and MMP-9 in patients with ME/CFS. IL-11 and MMP-9 levels in the serum of ME/CFS patients and healthy control subjects were quantified using commercial ELISA kits. The scatter plots represent serum **(A)** IL-11 levels in individual female patients (n=40; mean age 51 years) and age-matched control subjects (n=38; mean age 43 years) measured (***p<0.001). **(B)** the scatter plots represent serum MMP-9 levels in individual female patients (n=18; mean age 57 years) and age-matched control subjects (n=18; mean age 53 years; (****p<0.0001). The results were analyzed using Unpaired non-parametric Mann-Whitney test. Horizontal bars indicate the means of the data. Different patient cohorts for the IL-11 and MMP-9 were used due to the limited availability of samples for this pilot study.

There was a significant increase in serum levels of IL-11 in ME/CFS patients (mean 127 pg/ml; n=40) compared to healthy control subjects (mean 67 pg/ml), as shown in **Figure 1A** (n=38, p<0.001). Further, our results showed significantly increased levels of serum MMP-9 in ME/CFS patients (126 ng/ml; n=18) compared to healthy control subjects (17 ng/ml), as shown in **Figure 1B** (n=18, p<0.0001). Scatter graphs show individual data of IL-11 and MMP-9 in patients and control subjects. Horizontal bars in the scatter graphs are the means of the data. Different patient cohorts for IL-11 and MMP-9 were used due to lack of the same samples and limited numbers for this study.

**Figure 2:**
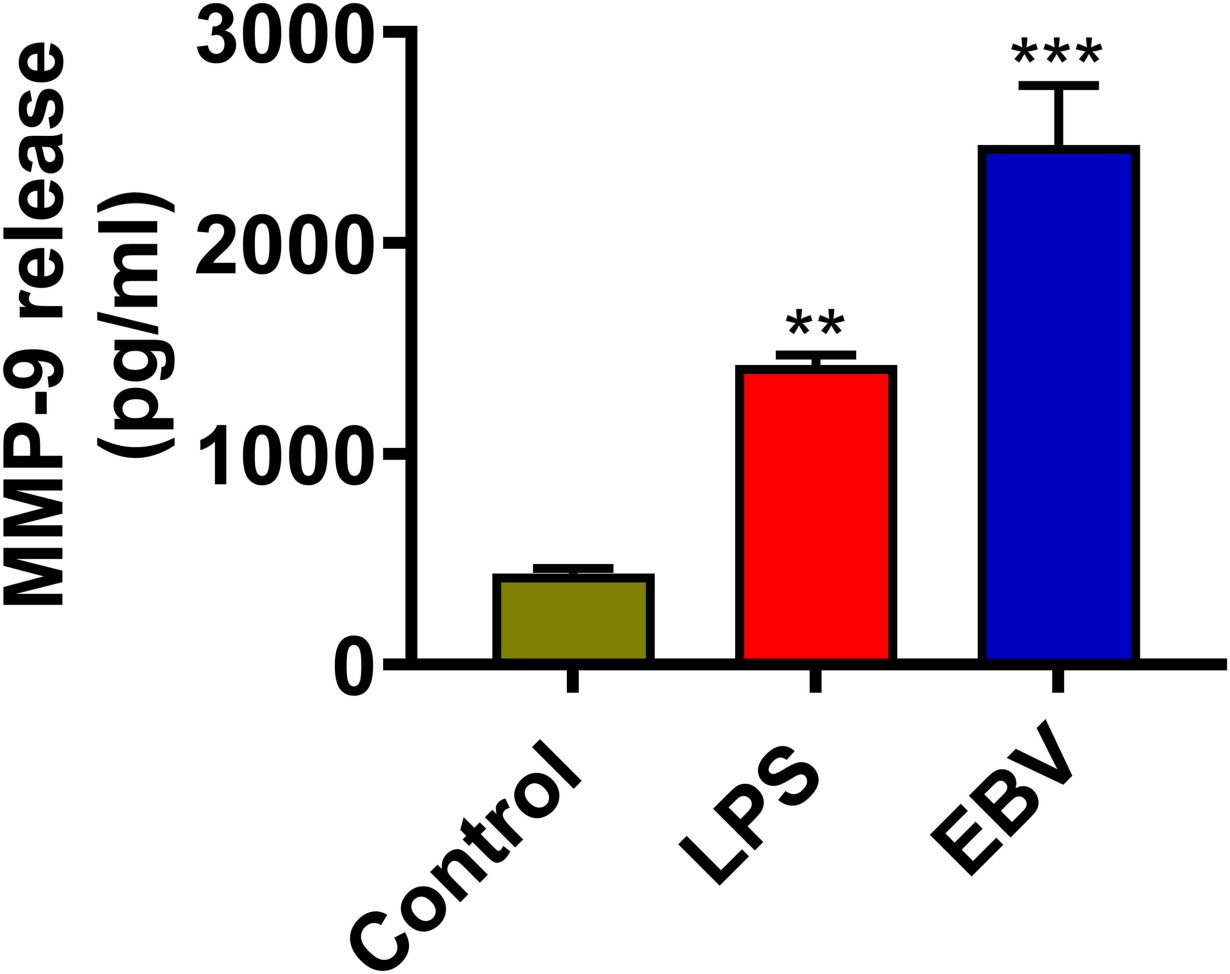
rEBV protein activates human MC and releases MMP-9 *in vitro*. MC (1x10^5^ cells/ml) were incubated with rEBV (100 ng/ml) protein for 24 hr in tissue culture plates at 37 °C. After the incubation was over, the culture supernatant media were collected by centrifugation and the levels of MMP-9 were quantified by ELISA (n=3). LPS (10 ng/ml) was used as a positive stimulant for MC. Both rEBV and LPS significantly released high levels of MMP-9 compared to untreated control cells (**p<0.01, ***p<0.001, one-way ANOVA and Tukey’s multiple comparisons test).

Next, we investigated if rEBV can activate MC to release MMP-9 *in vitro*. Incubation of human MC with rEBV (100 ng/ml) protein for 24 hr significantly released MMP-9 (mean 2,464 pg/ml) compared to untreated control cells (433 pg/ml; p<0.001). LPS used as a positive stimulant also significantly increased MMP-9 release (1,422 pg/ml) compared to untreated control cells (**Figure 2**; p<0.01 n=3).

## Discussion

In this pilot study, we found significantly elevated levels of IL-11 and MMP-9 in the serum of ME/CFS patients compared to age and gender-matched healthy control subjects. We also found that incubation of MCs with rEBV protein significantly increased the release of MMP-9 compared to untreated control cells in vitro, indicating a new source of MMP-9 from MC. Studies have shown that viral and other infections may be involved in the pathogenesis of ME/CFS (34, 35). High levels of IL-11 were reported in the respiratory tract of virally induced asthma (36). A recent review hypothesized that immunosenescence-associated age-dependent immunity decline could contribute initiate and maintaining fatigue in ME/CFS (37). Increased levels of IL-11 may contribute to the neurosenescence, inflammaging and immunosenescence, as recent reports demonstrated that IL-11 is the master regulator of aging in mice (38, 39). IL-11 could contribute to inflammaging, and pharmacologic inhibition of the IL-11 signaling pathway increases the lifespan and health span in mice (39). Another study also showed that blocking the age-related increase in IL-11 restores immune-metabolic homeostasis and extends health span and lifespan in mice (40). A recent review hypothesized that immunosenescence-associated age-dependent immunity decline could contribute to initiating and maintaining fatigue in ME/CFS (37). It has been shown that IL-11 activates nuclear factor-κB (NF-κB) and Janus kinase/signal transducers and activators of transcription (JAK/STAT) signaling pathway and mediates proinflammatory cytokine expression (19), which could contribute to the chronic inflammation in ME/CFS. Additional studies are needed to determine if inhibiting IL-11 would be useful to treat diseases or if it interferes with any physiological processes (39).

MMP-9 is crucial for synaptic plasticity, learning and memory, axonal regeneration and brain development (41), but also plays an important role in immune-associated CNS diseases (46). MMP-9-mediated blood-brain barrier (BBB) disruption is a hallmark of bacterial, viral, fungal, and parasitic CNS infections, facilitating immune cell infiltration and pathogen entry and exacerbating neuroinflammation and tissue damage (42, 43). High levels of plasma MMP-9 have been reported in mild cognitive impairment (MCI) and Alzheimer’s disease (AD) and may be involved in neuroinflammation and neurodegeneration (44). Another study reported that high levels of MMP-9 in cerebrospinal fluid (CSF) correlated with CNS infections (42). A recent study using a multiplexing custom assay for MMPs showed one patient cluster with higher levels of MMP-1, MMP-2 and MMP-10 as compared to another cluster of patients with ME/CFS (45).

The source of either IL-11 or MMP-9 in the serum of patients with ME/CFS is not presently known (15). IL-11 production in macrophages is regulated by several cytokines (17). Astrocytes are the main source of MMPs. Additionally, MMP-9 could be released from microglia (26). One study reported that the transformed human MC line secreted MMP-9 in response to phorbol 12-miristate 13-acetate (PMA) (27). Also, MC activation disorders have been frequently observed as a comorbid condition in patients with ME/CFS (28). EBV infection may be associated with the development of ME/CFS in some individuals (9, 10, 46). We found that rEBV protein activated MC to release high levels of MMP-9. We recently reported increased levels of MMP-9 in Long COVID patients, who share some similar symptoms with ME/CFS (30, 31), including infection-associated chronic immune exhaustion (47).

A combination of biomarkers may be useful for ME/CFS or a subcategory of patients (48–51). Since patients may have different underlying pathogenetic mechanisms (45). Moreover, symptomatology may vary potentially due to epigenetic mechanisms driven by environmental or stress triggers.

There are a number of limitations, including methodological biases, the different number of patients analyzed, the paucity of individual characteristics other than age and sex, and the differences in mean age group of the two patient groups. Moreover, the total number of samples analyzed was not large enough for both MMP-9 and IL-11 to make any subgroup analysis in ME/CFS. Also, the elevation of certain fluid biomarkers may vary depending on the disease status and the causes of the disease. Increased IL-11 level in this study could also be a compensatory protective mechanism in the ME/CFS patients and thus needs longitudinal analysis of these serum markers. We used n=3 in the in vitro experiments with MC since mature MC generation from CD34+ cells takes over 12 weeks in culture before they are ready to be used for stimulation experiments. The samples had been stored for over ten years at -80 °C, which may have altered the fragmentation of certain biomarkers. Finally, IL-11 was not secreted from MC in our study, suggesting that either it does not derive from MC or that there may be other triggers than rEBV.

## Conclusions

IL-11 and MMP-9 may be elevated in subsets of female ME/CFS individuals and could indicate unique pathologic mechanisms, possibly involving activation of mast cells by viral antigens.

## Data Availability

The original contributions presented in the study are included in the article/supplementary material. Further inquiries can be directed to the corresponding author.
